# Sulfur, sterol and trehalose metabolism in the deep-sea hydrocarbon seep tubeworm *Lamellibrachia luymesi*

**DOI:** 10.1186/s12864-023-09267-8

**Published:** 2023-04-05

**Authors:** Hong Shi, Lingwei Ruan, Zimeng Chen, Yifei Liao, Wenhao Wu, Linmin Liu, Xun Xu

**Affiliations:** 1 State Key Laboratory Breeding Base of Marine Genetic Resources, Key Laboratory of Marine Genetic Resources of Ministry of Natural Resources, Third Institute of Oceanography, Ministry of Natural Resources, Fujian Key Laboratory of Marine Genetic Resources, No. 178 Daxue Road, Xiamen, Fujian 361005 People’s Republic of China; 2 College of Marine Biology, Xiamen ocean vocational college, 361100 Xiamen, People’s Republic of China; 3 Co-Innovation Center of Jiangsu Marine Bio-Industry Technology, Jiangsu Ocean University, Lianyungang, 222005 People’s Republic of China; 4 School of Advanced Manufacturing, Fuzhou University, Fuzhou, 362200 People’s Republic of China; 5 Department of Biology and Guangdong Provincial Key Laboratory of Marine Biotechnology, Shantou University, Shantou, 515063 People’s Republic of China

**Keywords:** Adaptation to deep-sea chemosynthetic environment, Cold seep, Transcriptome, Sulfate, Cysteine, Cholesterol, Cycloartenol-C-24-methyltransferase, Trehalase, Fungi

## Abstract

**Background:**

*Lamellibrachia luymesi* dominates cold sulfide-hydrocarbon seeps and is known for its ability to consume bacteria for energy. The symbiotic relationship between tubeworms and bacteria with particular adaptations to chemosynthetic environments has received attention. However, metabolic studies have primarily focused on the mechanisms and pathways of the bacterial symbionts, while studies on the animal hosts are limited.

**Results:**

Here, we sequenced the transcriptome of *L. luymesi* and generated a transcriptomic database containing 79,464 transcript sequences. Based on GO and KEGG annotations, we identified transcripts related to sulfur metabolism, sterol biosynthesis, trehalose synthesis, and hydrolysis. Our in-depth analysis identified sulfation pathways in *L. luymesi*, and sulfate activation might be an important detoxification pathway for promoting sulfur cycling, reducing byproducts of sulfide metabolism, and converting sulfur compounds to sulfur-containing organics, which are essential for symbiotic survival. Moreover, sulfide can serve directly as a sulfur source for cysteine synthesis in *L. luymesi*. The existence of two pathways for cysteine synthesis might ensure its participation in the formation of proteins, heavy metal detoxification, and the sulfide-binding function of haemoglobin. Furthermore, our data suggested that cold-seep tubeworm is capable of de novo sterol biosynthesis, as well as incorporation and transformation of cycloartenol and lanosterol into unconventional sterols, and the critical enzyme involved in this process might have properties similar to those in the enzymes from plants or fungi. Finally, trehalose synthesis in *L. luymesi* occurs via the trehalose-6-phosphate synthase (TPS) and trehalose-6-phosphate phosphatase (TPP) pathways. The TPP gene has not been identified, whereas the TPS gene encodes a protein harbouring conserved TPS/OtsA and TPP/OtsB domains. The presence of multiple trehalases that catalyse trehalose hydrolysis could indicate the different roles of trehalase in cold-seep tubeworms.

**Conclusions:**

We elucidated several molecular pathways of sulfate activation, cysteine and cholesterol synthesis, and trehalose metabolism. Contrary to the previous analysis, two pathways for cysteine synthesis and the cycloartenol-C-24-methyltransferase gene were identified in animals for the first time. The present study provides new insights into particular adaptations to chemosynthetic environments in *L. luymesi* and can serve as the basis for future molecular studies on host-symbiont interactions and biological evolution.

**Supplementary Information:**

The online version contains supplementary material available at 10.1186/s12864-023-09267-8.

## Background

Cold seeps are areas of the ocean floor where leakage of hydrogen sulfide, methane, and other hydrocarbon-rich fluids occurs. These chemically and geologically diverse vent fields provide habitats for various vent fauna, including a single species of tubeworm, *Lamellibrachia luymesi* (Annelida: Siboglinidae). These tubeworms live only at shallow-water hydrocarbon seep vents in the Atlantic Ocean at depths of less than 1000 m [1].

These tubeworms lack a digestive tract and rely on symbiosis with chemoautotrophic bacteria [2]. They exchange gas with deep red plumes and take up sulfide from the environment by using extensions of their tubes that penetrate the seafloor sediment. Then, the sulfide is transported to the chemoautotrophic bacterial endosymbionts, belonging to Gammaproteobacteria, which live inside the bacteriocytes (specialized cells) of the trophosome (an organ produced by the host to house and protect its microbial partner). In return, these endosymbionts utilize the energy from sulfide oxidation to fuel the Calvin-Benson cycle. This respiratory process results in the production of organic carbon, which is provided to *L. luymesi*. This symbiosis has received much attention, and studies have examined the energy metabolism, host-microbe interactions, and adaptation to deep-sea chemosynthetic environments to reveal unique mechanisms associated with chemical ecology and metabolic evolution.

To date, most studies of tubeworms and their endosymbionts have focused on the hydrothermal vent tubeworm *Riftia pachyptila* (Vestimentifera), including studies on the metabolism of sulfide [3–7], hydrogen [8], nitrogen [9–12], and carbon [13]. The *L. luymesi* genomic sequences were recently published [14], and the mechanisms related to nutrition mode, haemoglobin evolution, immunity function, longevity, and the cell cycle were discussed. Moreover, we have completed the *L. luymesi* transcriptome sequencing and assembly analysis. The availability of this large set of transcriptomic and genomic data from *L. luymesi* facilitates further exploration of the adaptation of deep-sea fauna to chemosynthetic environments.

Of particular interest has been the ability of hydrocarbon seep tubeworms to cope with toxic levels of hydrogen sulfide and eliminate sulfate, the primary waste product of chemoautotrophic sulfide oxidation. Tubeworms rely on sulfide for metabolism; hydrogen sulfide and oxygen are delivered to chemoautotrophic bacteria by haemoglobin [3, 14–17]. Moreover, more haemoglobin B1 gene copies were recently found in the genome of *L. luymesi* than in other siboglinids, which might indicate increased sulfide-binding capacity to fulfil symbiosis [18]. On the other hand, symbionts oxidize sulfide to produce energy for carbon fixation and release sulfate and hydrogen ions as byproducts [6, 7]. *L. luymesi* was predicted to eliminate these byproducts across its roots to conserve energy and ensure sulfide supply. The elimination of metabolic waste products is vital for *L. luymesi*. However, the oxidized oxyanion sulfate is also the most available form of sulfur in nature, and detailed knowledge of how *L. luymesi* activates sulfate and incorporates it into diverse metabolites is unknown.

Deep-sea animals live under high pressure. One of the ways in which they adapt to their harsh environment might result from the unique properties of the cellular membrane. Sterols are essential components of the membranes of all eukaryotic organisms, controlling membrane fluidity and permeability [19]. Moreover, some cyclic triterpenoid lipids, such as steranes (degraded and saturated derivatives of sterols), are essential and have remained stable through deep geological time. They have been proposed as molecular fossils, recording the evolution of organisms even in the absence of physically preserved fossils [20]. Indeed, sterol biosynthesis is a multistep process catalysed by a series of enzymes. Animals (e.g., mammals) can de novo synthesize cholesterol from low molecular weight precursors such as acetate [21]. Nevertheless, several marine invertebrates (e.g., *Bathymodiolus* mussels) are exceptional in their ability to synthesize cholesterol de novo by themselves, converting exogenous sterols to cholesterol [22]. Interestingly, a recent study revealed that *L. luymesi* lacks many genes essential for amino acid biosynthesis and might depend mainly on endosymbionts for nutrition [14]. However, information on the sterol biosynthetic pathway in *L. luymesi* is lacking.

The sugar trehalose (α-D-glucopyranosyl (1, 1)-α-D-glucopyranoside), a nonreducing disaccharide of glucose, is found widely but not abundantly in nature [23, 24]. Its functions proposed by Colaço and Roser in 1995 are myriad and include water replacement, glass transformation, and chemical stability [25]. Thus, this sugar can act as a stress protectant in biological systems as it interacts with and directly protects membranes and proteins from the damage caused by physiological and environmental changes [26]. For this reason, many organisms accumulate trehalose to adapt to environmental extremes [27]. Additionally, trehalose also acts as an energy reserve in nematodes and insects. It appears to function as a significant circulating blood sugar [28, 29]. Based on the importance of trehalose metabolism for extreme adaptation in animals, we aimed to characterize enzymes involved in the synthesis and hydrolysis of trehalose.

Here, we report the assembly of the cold-seep *Lamellibrachia luymesi* transcriptome. By combining transcriptomic and genomic data, we further investigated the adaptation to deep-sea chemosynthetic environments in *L. luymesi* with special attention to sulfur, sterol, and trehalose metabolism pathways. The novel candidate genes identified in these pathways could be associated with the extraordinary adaptation and symbiotic associations of tubeworms. These results provide new insights into adaptations to chemosynthetic environments in *L. luymesi*.

## Results and discussion

### De novo assembly and functional annotation of the *L. luymesi* transcriptome

**Fig. 1:**
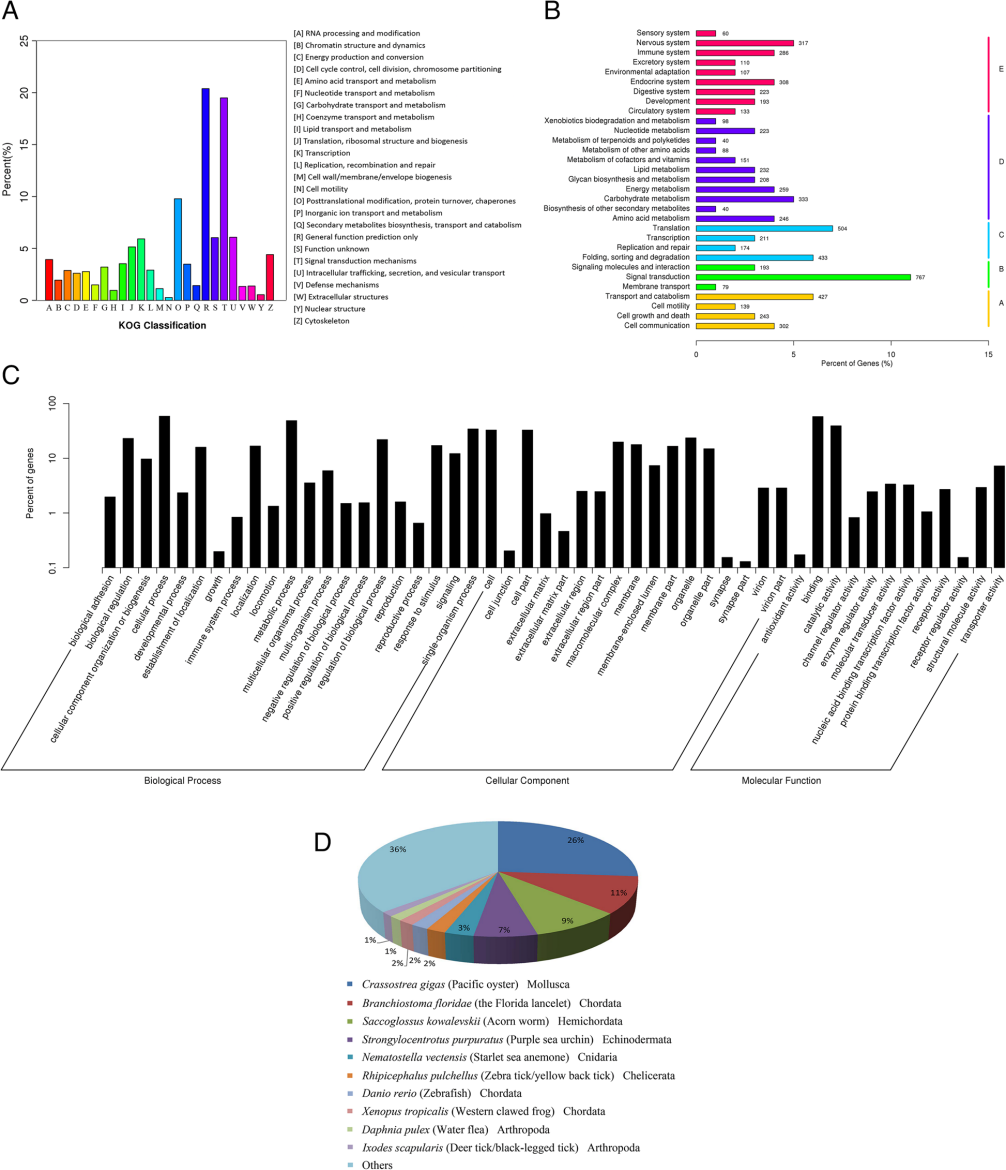
A summary of the functional annotation of the *L. luymesi* transcriptome. Functional classification of transcripts annotated by **A** KOG; **B** KEGG; **C** GO. **D** Species distribution of annotated transcripts

The mitochondrial cytochrome c oxidase subunit I (COI) gene sequence of the specimen shared 100% identity with the corresponding *L. luymesi* sequences from the Gulf of Mexico (GenBank accession no. GU059168.1). We produced 6.86 Gbp of clean data from the tissue sample. Over 96% of the clean Illumina reads exceeded Q20, indicating the high quality of the sequencing data. The raw sequencing data have been submitted to the Short Read Archive (SRA) of NCBI under accession number SRR17999638. The final transcriptome generated from Trinity de novo assembly contained 79,464 transcript sequences. The lengths of the transcripts ranged from 200 to 13,725 bp, with an average length of 1047 bp and an N50 value of 1906 bp. The statistics for the data output and de novo assemblies are summarized in Table 1. The length distribution of all the transcripts and unigenes is shown in Figure S1. In total, 21,300 (43.24%) unigenes had at least one significant hit in the NCBI nonredundant (nr), Swiss-Prot, KO, PFAM, GO, and KOG databases and the NCBI nucleotide database. Over half of the dominant transcripts had no match among proteins in any public database. The functional annotation results are shown in Table 1. The KOG functional categories and KEGG pathways for the annotated unigenes are shown in Fig. 1A and B, respectively. There were 16,887 (34.28%) nr-annotated unigenes assigned to major Gene Ontology (GO) categories, i.e., “Biological Process”, “Cellular Component”, and “Molecular Function” (Fig. 1C). ESTscan and BLAST search of all the databases mentioned above resulted in the prediction of 30,781 protein-coding transcripts (Table 1). The best hit for most of the annotated unigenes (4412 out of 16,887, 26.1%) was *Crassostrea gigas* in the nr database (Fig. 1D). This result might be attributed to annelids and molluscs sharing a sister phylogenetic relationship [30] and to the availability of molecular resources for marine annelids being scarcer than that for other phyla.

**Table 1:** Summary of assembling and functional annotation of *L. luymesi* transcriptome

**De novo assembly by Trinity**	
** Total base (bp)**	68,590,831
** Total number of transcripts**	79,464
** Number of unigenes**	49,254
** Mean length of transcripts (bp)**	1,047
** N50 (bp)**	1,906
** Transcripts size range (bp)**	200–13,725
**Functional annotation**	
** Total number of Unigenes annotated by public databases**	49,254
** NCBI non-redundant (nr) database (e-value < 1e-5)**	16,887
** SwissProt (e-value < 1e-5)**	13,654
** PFAM**	15,753
** Gene Ontology**	16,254
** KOG**	9,211
**Coding sequence prediction**	
** CDS predicted from BLAST results**	17,055
** CDS predicted by ESTScan**	13,726
** Total CDS predicted**	30,781
** Mean length of CDS (aa)**	196

### Sulfur metabolism and cysteine biosynthesis

*L. luymesi* acquires sulfide from the sediment and then supplies sulfide to endosymbiotic bacteria to produce energy for carbon fixation. While *L. luymesi* obtains its nutrition from symbionts, they must eliminate or transform sulfate byproducts.

**Fig. 2:**
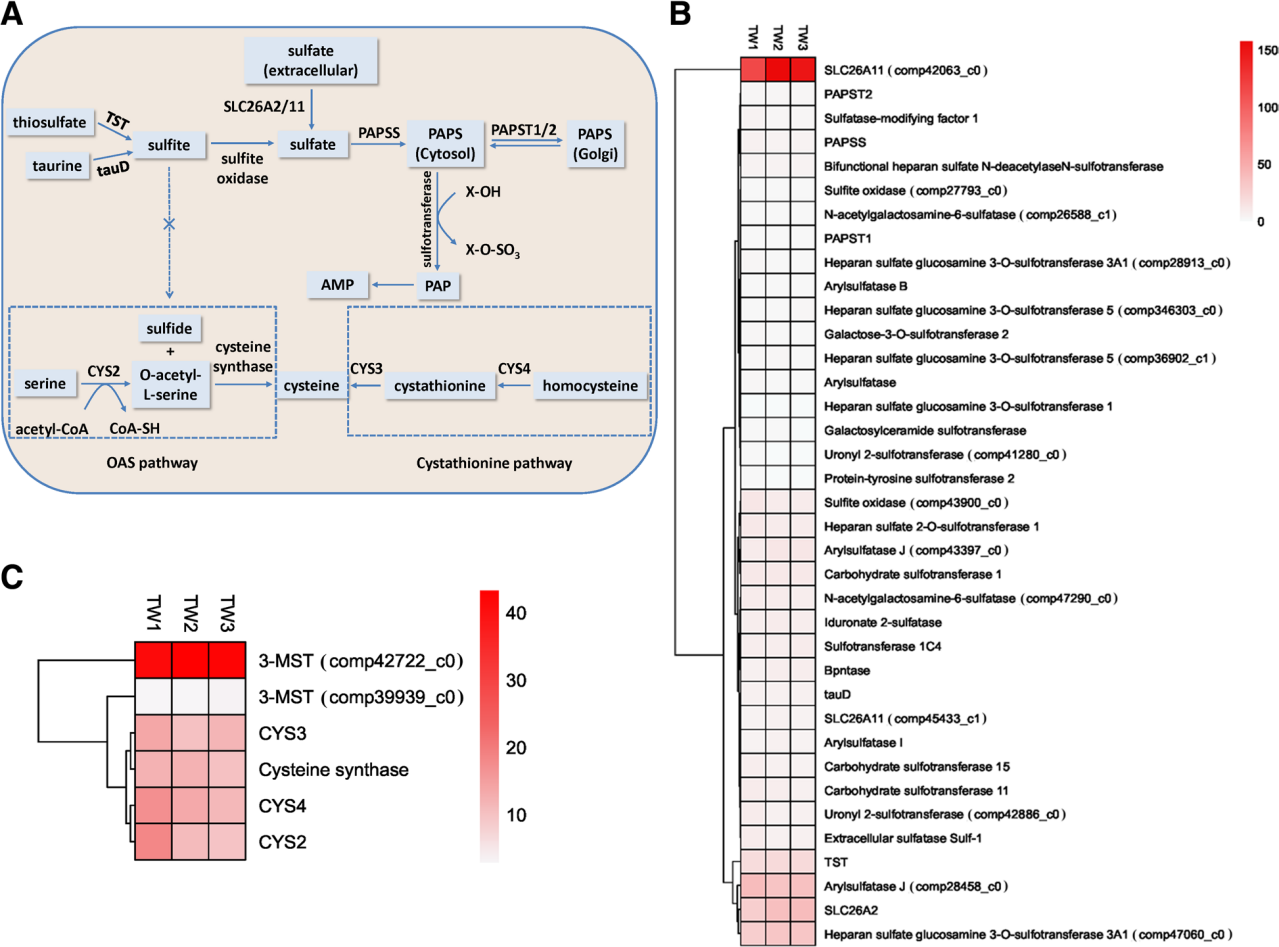
**A** Scheme of *L. luymesi* sulfation pathways and biosynthetic pathways for cysteine. TST, thiosulfate sulfurtransferase; tauD, alpha-ketoglutarate-dependent taurine dioxygenase; SLC26A2, sulfate transporter; SLC26A11, sodium-independent sulfate anion transporter; PAPSS, bifunctional 3'-phosphoadenosine 5'-phosphosulfate synthase; PAPST1/2, adenosine 3'-phospho 5'-phosphosulfate transporter 1/2; CYS2, probable serine-O-acetyltransferase cys2; CYS3, putative cystathionine gamma-lyase 2; CYS4, cystathionine beta-synthase. **B** Heatmap of FPKM expression values for the annotated genes in *L. luymesi* sulfation pathway from three individual samples (TW1, TW2 and TW3). **C** Heatmap of FPKM expression values for the annotated genes in *L. luymesi* biosynthetic pathways for cysteine. FPKM values were listed in Table S4

**Fig. 3:**
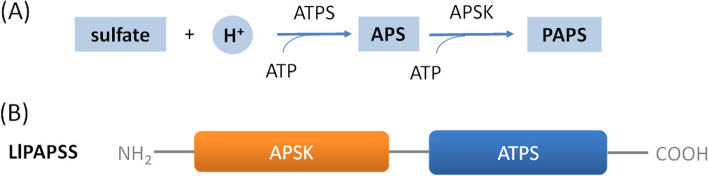
PAPS synthesis in *L. luymesi*. **A** PAPS synthesis pathway. **B** The domain formation of *L. luymesi* PAPSS, a fused gene encoding a protein with two conserved domains. APS, adenosine 5'-phosphosulfate; PAPS, bifunctional 3'-phosphoadenosine-5'-phosphosulfate; PAPSS, PAPS synthase; APSK, APS kinase; ATPS, ATP sulfurylase

The present study found that sulfation pathways are present in *L. luymesi*. We have depicted the *L. luymesi* sulfation pathways in Fig. 2A and Table S1. The FPKM (fragments per kilobase million) values of genes in this pathway are shown in Fig. 2B. Several sulfate transporters and sulfite oxidases are responsible for cellular sulfate uptake, followed by the two-step enzymatic sulfate activation performed by the bifunctional 3'-phosphoadenosine-5'-phosphosulfate synthase (PAPSS). PAPSS in *L. luymesi* possesses both ATP sulfurylase (SUL) and APS kinase (KIN) activities (Fig. 3). This fusion probably increases the catalytic efficiency [31, 32]. The product PAPS, as the activated sulfate group donor, is either used directly by cytoplasmic and nuclear sulfotransferases or shuttled to the Golgi apparatus to serve a multitude of Golgi-residing carbohydrate and protein sulfotransferases. Due to the substrate diversity, sulfotransferases and sulfatases form large multigene families. These enzymes, which participate in sulfation and desulfation of bioorganic compounds, are essential for cellular activity in animal cells, such as extracellular communication, inflammation, and lymphocyte homing, and play a vital role in a functional endocrine system [33–36]. Furthermore, cytoplasmic adenosine 3′,5′-bisphosphate (PAP), the otherwise toxic byproduct of sulfation, is removed and degraded to AMP by phosphate 3'(2'),5'-bisphosphate nucleotidase. Therefore, sulfate activation could be an important detoxification pathway in *L. luymesi* to promote sulfur cycling, reduce sulfate, and convert sulfur compounds to sulfur-containing organics essential for the tubeworm.

Additionally, the heat map displays the relative expression levels of two sulfate transporter genes (sulfate transporter and sodium-independent sulfate anion transporter, also known as SLC26A2 and SLC26A11 in human, respectively) are particularly high, compared to that of other genes in this pathway (Fig. 2B). SLC26A11 was sensitive to the anion exchange inhibitor DIDS and can specifically mediate Cl^−^/HCO_3_^−^ exchange, remarkable co-localized with H^+^-ATPase in human cells [37, 38]. On the other hand, Dattagupta et al. previously proposed a model to indicate *L. luymesi* primarily eliminated the sulfate mainly with transporters coupled with bicarbonate uptake [6]. This model depends on an anion exchanger that is sensitive to DIDS and exchanges sulfate ions for either Cl^−^ or HCO_3_^−^. Thus we speculated that SLC26A2 and SLC26A11 might be the potential sulfate-bicarbonate exchangers which are highly expressed in *L. luymesi*. This result provided evidence and further support that sulfation pathway might be involved in the detoxification for promoting sulfur cycling and reducing byproducts.

*Sulfite reductase* is a flavoprotein complex that catalyses the reduction of sulfite to sulfide in the cysteine and methionine biosynthesis pathway. Four transcripts of sulfite reductase (three alpha and one beta component) were found in the cold seep mussel *Bathymodiolus platifrons* [39]. It is worth noting that we failed to find any gene that could encode a sulfite reductase homologue. The transcriptome and genome sequence analyses did not have 100% coverage, so we cannot completely discount the possibility that the gene is present. However, because *L. luymesi* lives in sulfide-rich subsurface sediment zones, the existence of sulfite reductase might not be necessary for symbiotic survival.

In addition, sulfur is also a constituent of the amino acid *cysteine*. Interestingly, in the present study, the transcriptome analysis revealed four genes encoding proteins possibly involved in two pathways for cysteine synthesis in *L. luymesi*, namely, cystathionine gamma-lyase (CYS3), cystathionine beta-synthase (CYS4), serine-O-acetyltransferase (CYS2), and cysteine synthase (Table S1). Thus, sulfide can be a direct sulfur source for cysteine synthesis in *L. luymesi*.

In general, two different routes for cysteine biosynthesis have been described in animals and plants/bacteria. Cysteine biosynthesis begins with the amino acid serine in animals. Sulfur is derived from methionine, which is converted to homocysteine. CYS4 combines homocysteine and serine to form the asymmetrical thioether cystathionine. CYS3 further converts cystathionine to cysteine and alpha-ketobutyrate. This pathway is called the cystathionine pathway. Moreover, in plants and bacteria, cysteine biosynthesis also starts from serine, which is converted to O-acetylserine (OAS) by CYS2. Then, cysteine synthase, using sulfide sources, converts this ester to cysteine, releasing acetate [40, 41] (Fig. 2).

**Fig. 4:**
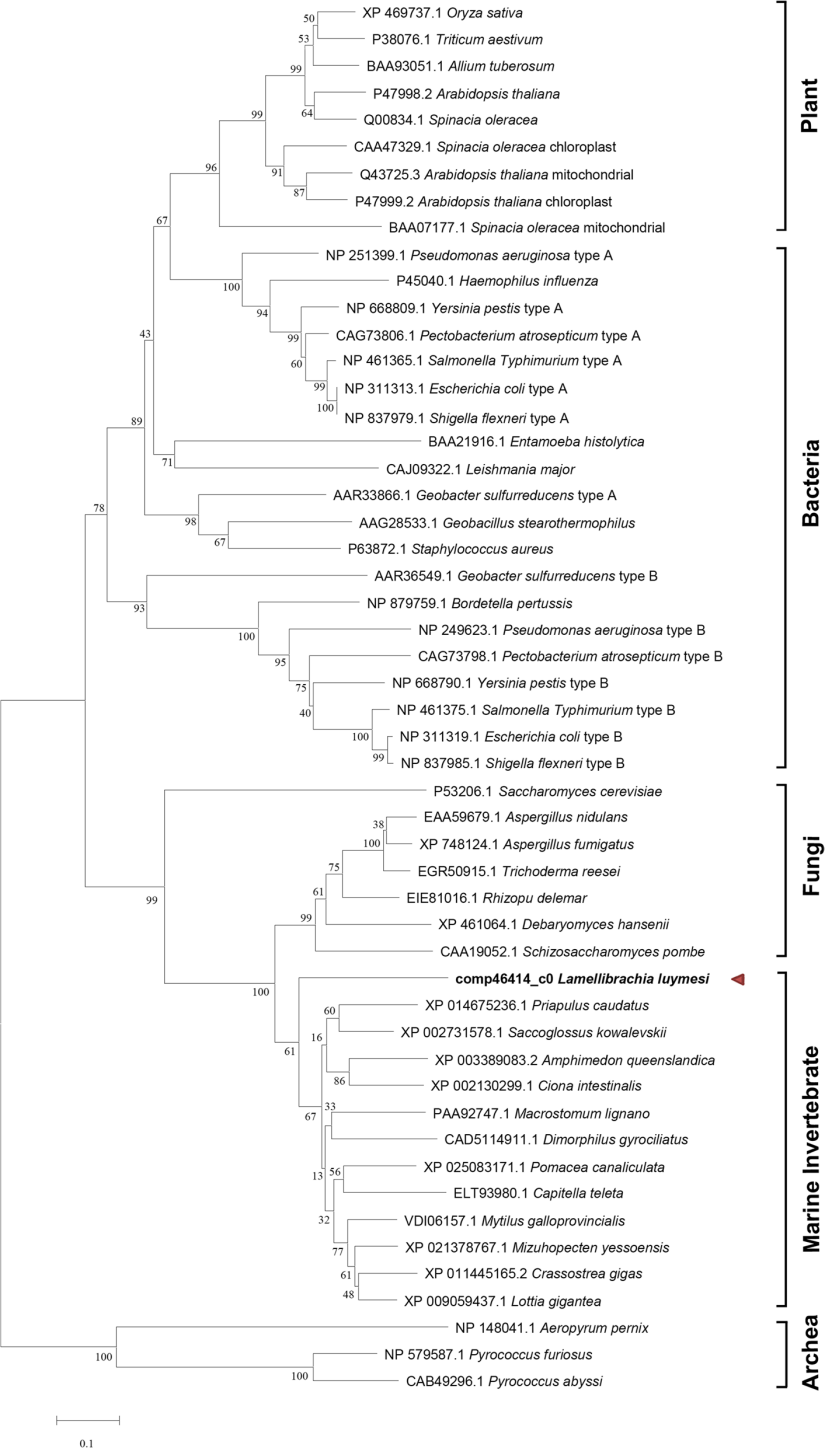
Phylogenetic tree of CS sequences. *L. luymesi* CS is highlighted in bold

To the best of our knowledge, this is the first report showing that two pathways for cysteine synthesis are present in animals. In the OAS pathway, *cysteine synthase* (also known as O-acetylserine sulfhydrylase, cysK) is a pyridoxal 5'-phosphate-dependent enzyme that catalyses the final step. Cysteine synthase of *L. luymesi* (LlCS) is predicted to encode a protein of 374 amino acids, with a subunit molecular mass of 40.37 kDa. Phylogenetic analysis showed that the LlCS separated from other marine invertebrate to form an independent evolutionary branch and aligned more closely with CSs of fungi than CSs from bacteria, plants, and archaea (Fig. 4). To date, the existence of two pathways for cysteine synthesis has been demonstrated in fungi, such as the filamentous fungus *Aspergillus nidulans*, which has been reported to have the richest repertoire of sulfur metabolic options [42]. Analysis of the sulfur amino acid metabolism in *L. luymesi* demonstrated that it shares features of plants, bacteria and animal systems in cysteine interconversion. This characteristic highlights that the cysteine synthesis pathway plays a vital role in *L. luymesi*. Cysteine is involved in the structure, stability, and catalytic functions of many proteins and contributes to the antioxidant activity of glutathione [43]. Cysteine biosynthesis is critical for heavy metal and metalloid resistance, such as arsenic and cadmium resistance [44]. More importantly, cysteine residues in haemoglobin determine the sulfur binding capabilities that permit tubeworms to live in chemosynthetic conditions [18, 45].

Whether *serine-O-acetyltransferase* is present in *L. luymesi* might be controversial. Published genome analysis showed that serine-O-acetyltransferase (also known as cysE) was present in symbionts but absent in the Lamellibrachia host [18]. This study found that a probable serine-O-acetyltransferase (LlCYS2) was annotated in the *L. luymesi* transcriptome and genome. Moreover, sequence analysis revealed that LlCYS2 shares significant similarity with Cys2 in *Schizosaccharomyces pombe.* As noted previously, *S. pombe* Cys2 is a serine O-acetyltransferase specifically essential for cysteine biosynthesis [44, 46]. Therefore, LlCYS2 is more likely a serine O-acetyltransferase involved in the OAS pathway for cysteine biosynthesis in *L. luymesi*.

### Biosynthetic pathways for sterols

Sterols are crucial for the functioning of most eukaryotic cells and the structure of their membranes and serve as precursors to signalling and hormone molecules to control developmental processes [47, 48].

**Fig. 5:**
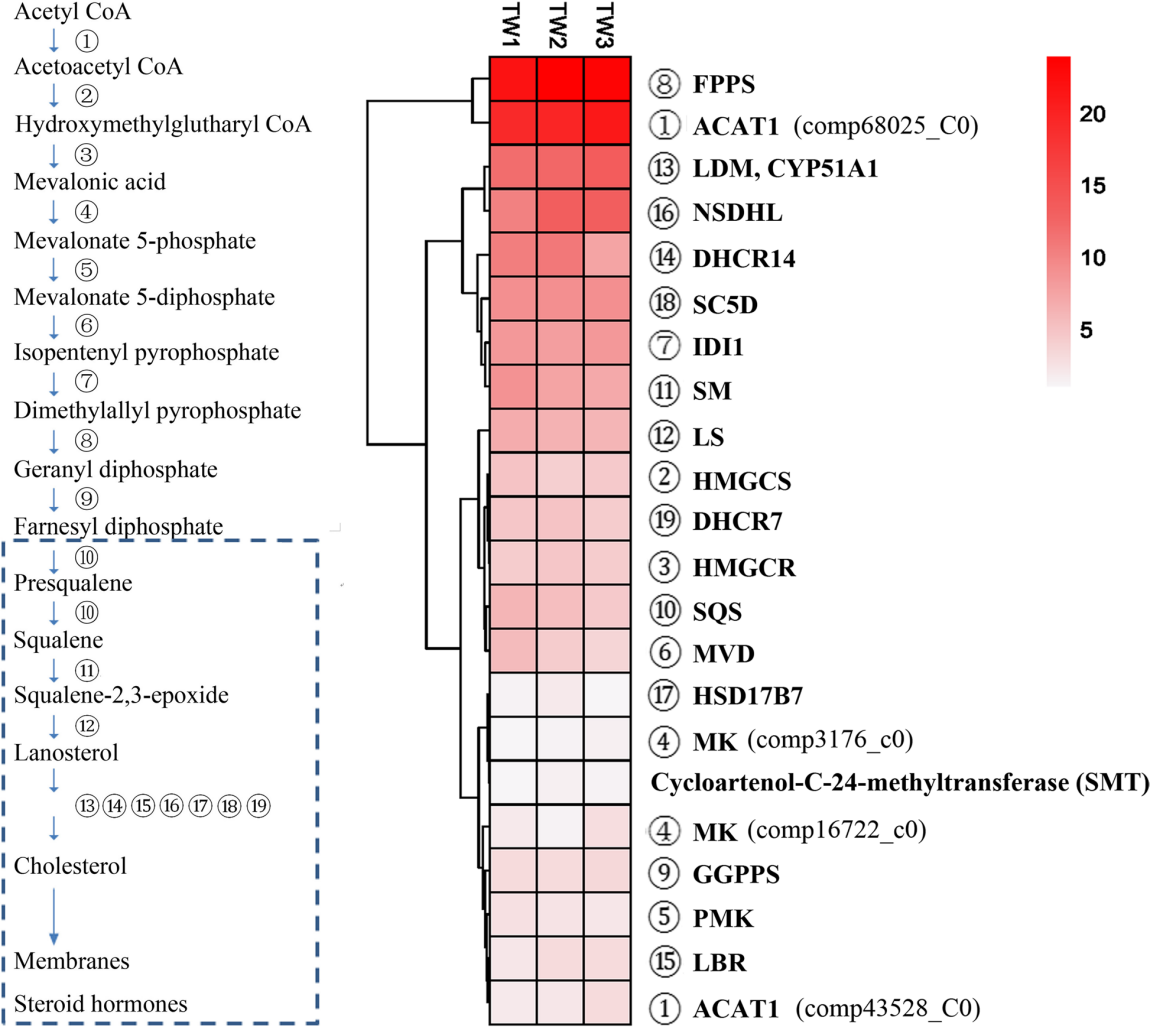
**A** Overview of cholesterol synthesis pathway in *L. luymesi*. Circles represent the following enzymes: 1, acetyl-CoA acetyltransferase; 2, hydroxymethylglutaryl-CoA synthase; 3,3-hydroxy-3-methylglutaryl-coenzyme A reductase; 4, mevalonate kinase; 5, promyelvalonate kinase; 6, diphosphomevalonate decarboxylase; 7, isopentenyl-diphosphate delta-isomerase; 8, farnesyl pyrophosphate synthase; 9, geranylgeranyl pyrophosphate synthase; 10, squalene synthase; 11, squalene monooxygenase; 12, lanosterol synthase; 13, lanosterol 14-alpha demethylase; 14, delta(14)-sterol reductase; 15, lamin-B receptor; 16, sterol-4-alpha-carboxylate 3-dehydrogenase; 17, 3-keto-steroid reductase; 18, lathosterol oxidase; 19, 7-dehydrocholesterol reductase. *C. elegans* lacks the branch inside the dashed box. **B** Heatmap of FPKM expression values for the annotated genes in *L. luymesi* cholesterol synthesis pathway from three individual samples (TW1, TW2 and TW3). FPKM values were listed in Table S4

Mammals synthesize squalene and various sterols from low-molecular-weight precursors such as acetate via mevalonate. However, other animals exclusively depend on dietary sterols due to the complete or partial absence of a cholesterol biosynthetic pathway, such as insects, nematodes [49, 50], the annelid *Lumbricus terrestris* [51, 52], and some marine invertebrate crustaceans [53–56]. For example, *C. elegans* expresses predicted homologues of the enzymes that produce the initial intermediates of the mammalian sterol biosynthetic pathway up to farnesyl diphosphate but cannot synthesize either squalene or lanosterol [49]. Deep-sea *Bathymodiolus* mussels by themselves cannot synthesize cholesterol de novo and depend mainly on endosymbionts for nutrition [22]. There are no genes encoding enzymes associated with upstream steps of the cholesterol biosynthesis pathway in the Pacific oyster *Crassostrea gigas* [57]. In the present study, 19 enzymes involved in biosynthetic pathways for sterols were identified (Fig. 5) (Table S2). The annoated enzymes in this pathway are activated (Fig. 5B), and farnesyl pyrophosphate synthase (FPPS) displays the highest expression. This may be attributed to its substrate farnesyl diphosphate which is a branching point to the biosynthesis of vital classes of molecules, such as ubiquinone or dolichol. These results indicated that *L. luymesi* could synthesize cholesterol from acetate or mevalonate via squalene and lanosterol by themselves and suggested that *L. luymesi* might possess cholesterol as its principal sterol.

In this pathway, a *lanosterol 14-alpha demethylase* (also known as cytochrome P450 family 51 subfamily A member 1, CYP51A1) transcript which has relatively high expression was detected in the transcriptome of *L. luymesi* (Table S2). It is involved in the conversion of lanosterol to 4,4-dimethylcholesta-8(9),14,24-trien-3β-ol. This demethylation step is the initial checkpoint in the transformation of lanosterol to other sterols widely used within the cell [58]. CYP51A1 is studied primarily in fungi, where it plays an essential role in mediating membrane permeability [59]. CYP51 is an essential enzyme in sterol biosynthetic pathways and the only and evolutionarily oldest P450 gene family with catalytically identical orthologues in different biological kingdoms [58, 60]. The CYP51 proteins have low sequence similarity across phyla but a highly conserved catalytic function. This requires CYP51s have a typical configuration of their substrate-binding pockets and specific amino acid conservation. Most of the conserved CYP51 residues are clustered into six regions, representing substrate recognition sites (SRSs) 1–5 and surrounding the heme-coordinating Cys [60, 61]. The most conserved, SRS1 and SRS4, are regarded as the CYP51 signature. SRS1 forms the upper surface of a P450 substrate-binding cavity. SRS4 is located in the C-terminal part of the P450 I-helix, forming the right wall of the distal surface of the substrate-binding cavity.

**Fig. 6:**
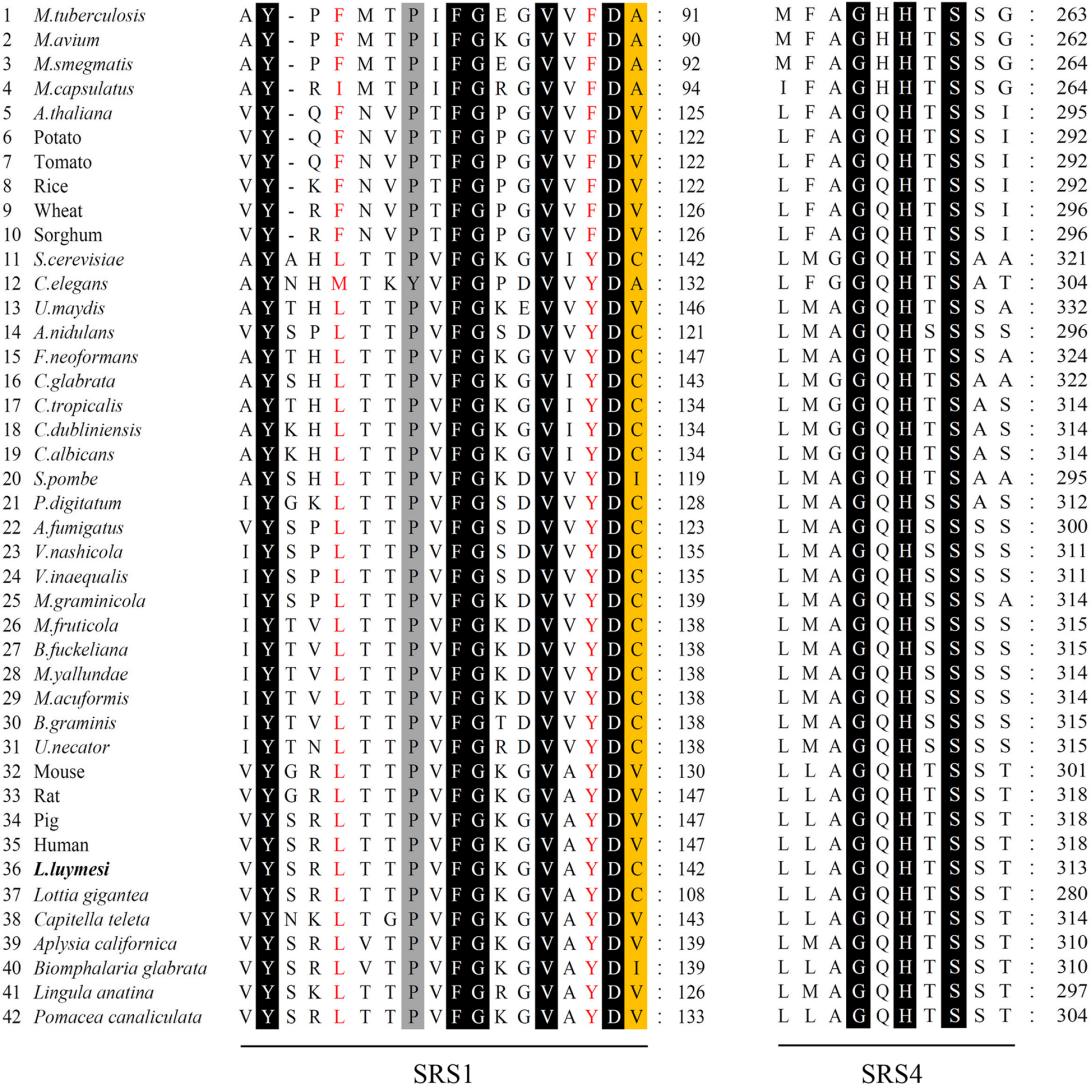
Sequence alignment of 42 CYP51 family members from different biological kingdoms [bacteria (1–4), plants (5–10), fungi (11, 13–31) and animals (12, 32–42)] in CYP51 substrate recognition sites (SRS) 1 and 4; 100% and more than 95% conserved residues are shaded in black and grey, respectively. Phyla-specific residues are highlighted in red or shaded in yellow. The accession number of sequences can be found in Supplemental data file 1

SRS1 and SRS4 were found in *L. luymesi* CYP51A1 (LlCYP51A1) (Fig. 6). LlCYP51A1 SRS1 shares high identity with the human orthologue with only a one-residue difference (residue C). Interestingly, this different residue, C141, was ubiquitous in fungi, and in animals, V is always present at this position, except in one sequence from *Biomphalaria glabrata* (I). Additionally, the residue F/L in SRS1 is phylum-specific (F in plant and L in animal/fungal CYP51). The phylum-specific residue is used to predict preferred substrates (mono- or dimethyl at C4) of newly identified sterol 14α-demethylases. Thus, we speculated that *L. luymesi* CYP51A1 probably has the physiological substrate C-4 dimethylated and can metabolize four substrates (lanosterol, 24, 25-dihydrolanosterol, 24-methylenedihydrolanosterol, and obtusifoliol) [62].

**Fig. 7:**
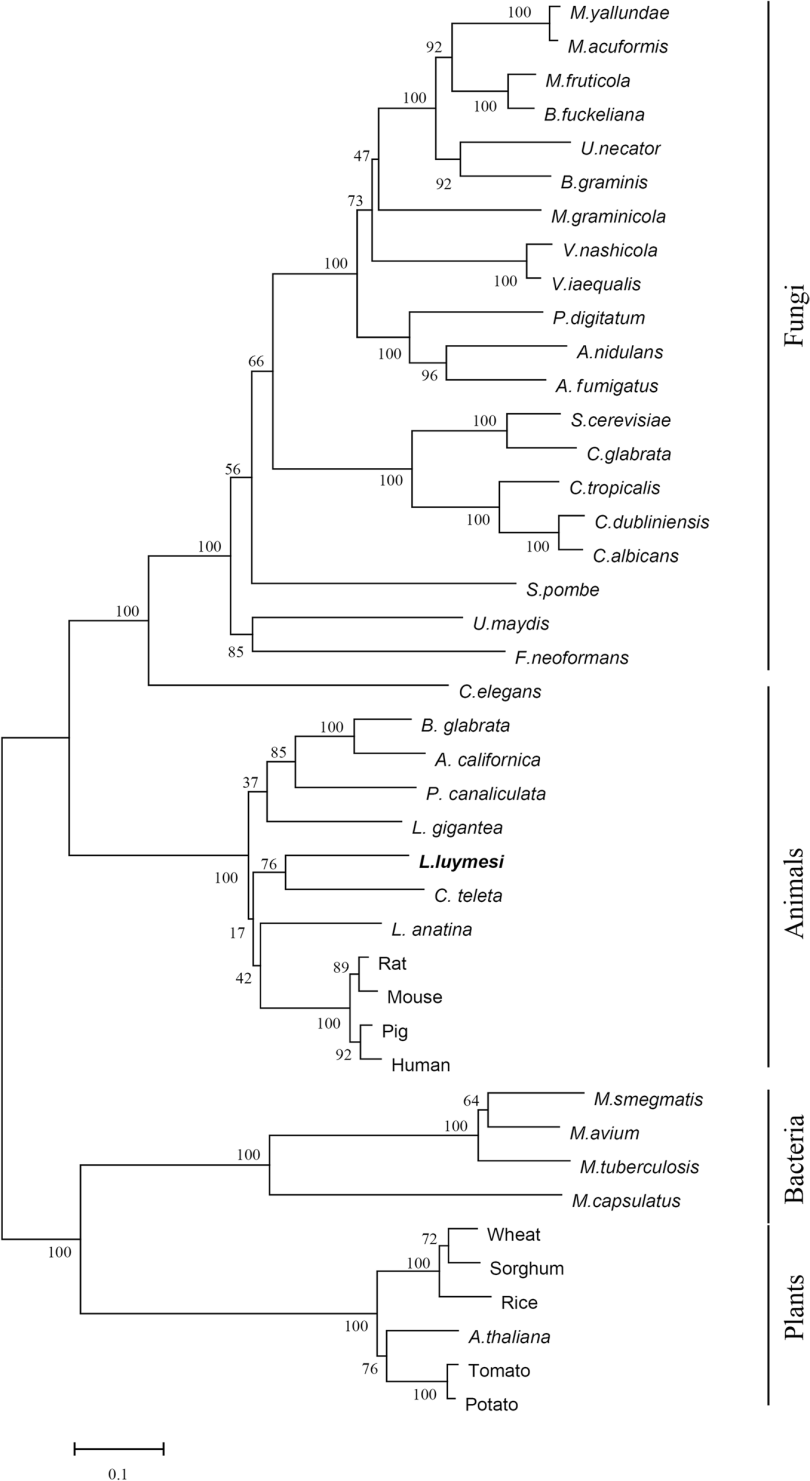
Phylogenetic tree of 42 CYP51 family members with 1000 bootstrap replications. *L. luymesi* CYP51A1 is highlighted in bold. The accession number of sequences can be found in Supplemental data file 1

Phylogenetic analysis suggested that *L. luymesi* CYP51A1 is homologous to other CYP51s. It shared 62% amino acid identity with the human CYP51A1 sequence. CYP51 proteins in fungi and animals clustered within an independent clade, while those in bacteria and plants clustered in another clade on the phylogenetic tree. *L. luymesi* CYP51A1 was assigned to the animal group, and *Capitella teleta* CYP51 formed an independent branch (Fig. 7).

Unexpectedly, a transcript of *cycloartenol-C-24-methyltransferase*, which catalyses the transfer of the methyl group from S-adenosyl-methionine to the C-24 of cycloartenol to form 24-methylene cycloartenol, was identified in *L. luymesi*. There are two copies of the gene in the *L. luymesi* genome. Cycloartenol is an important triterpenoid of the sterol class, which is found in plants. It is the starting point for the synthesis of almost all plant steroids [48], making them chemically distinct from the steroids of fungi and animals that are instead produced from lanosterol. Cycloartenol-C-24-methyltransferase is a sterol C24-methyltransferase (SMT), which are unique to fungi, plants, and protozoa but are not synthesized by animals, as reported previously [63]. Thus, this sequence was identified in animals for the first time.

Alignment against the NCBI database showed that the genes from only *Capitella teleta* and *Thecamonas trahens* ATCC 50,062 were homologous to *L. luymesi* SMT, and they shared 52% and 47% sequence identity, respectively. Most *L. luymesi* SMT orthologues were derived from Ostreococcus and higher plants. Plant sterols in animal tissues were detected previously. The analysis of sterol composition obtained by gas chromatography and mass spectrometry showed the presence of plant sterols in the tissues of shallow tropical sponges and cnidarians, as well as their deep-sea counterparts [64]. Significantly, it was proven that most sponges could synthesize sterols de novo and transform cycloartenol and lanosterol, the sterol precursors of photosynthetic and nonphotosynthetic organisms, respectively, into 4,4,14-demethyl sterols. Symbiotic cyanobacteria found in many sponges are not involved in the observed biosynthesis [65, 66]. Thus, our data suggested that *L. luymesi* should be capable of incorporating and transforming cycloartenol to unconventional sterols, while the critical enzyme involved in this process might have similar properties to those in plants. However, the source of cycloartenol in *L. luymesi* is unclear. It might be derived from some photosynthetic symbiotic organism and ingested by tubeworm. As another possibility, the *L. luymesi* SMT arose through lateral gene transfer from the symbiont also deserves consideration. It was predicted that the lipids found in *L. luymesi* might differ significantly from those found in other living organisms. The unique sterol metabolism in *L. luymesi* might play a vital role in membrane structure fluidity and developmental regulation associated with adaptation to a unique environment. This finding might also indicate a candidate gene for research on biological evolution.

#### Trehalose metabolism

Trehalose is found in various organisms, including bacteria, yeast, fungi, insects, invertebrates, and plants, but it is absent in vertebrates. It plays several important physiological roles as a carbon source, reserve carbohydrate, compatible solute, nutritional and environmental stress protector, metabolic regulator, and even signalling transducer or virulence factor, among others [67].

**Fig. 8:**
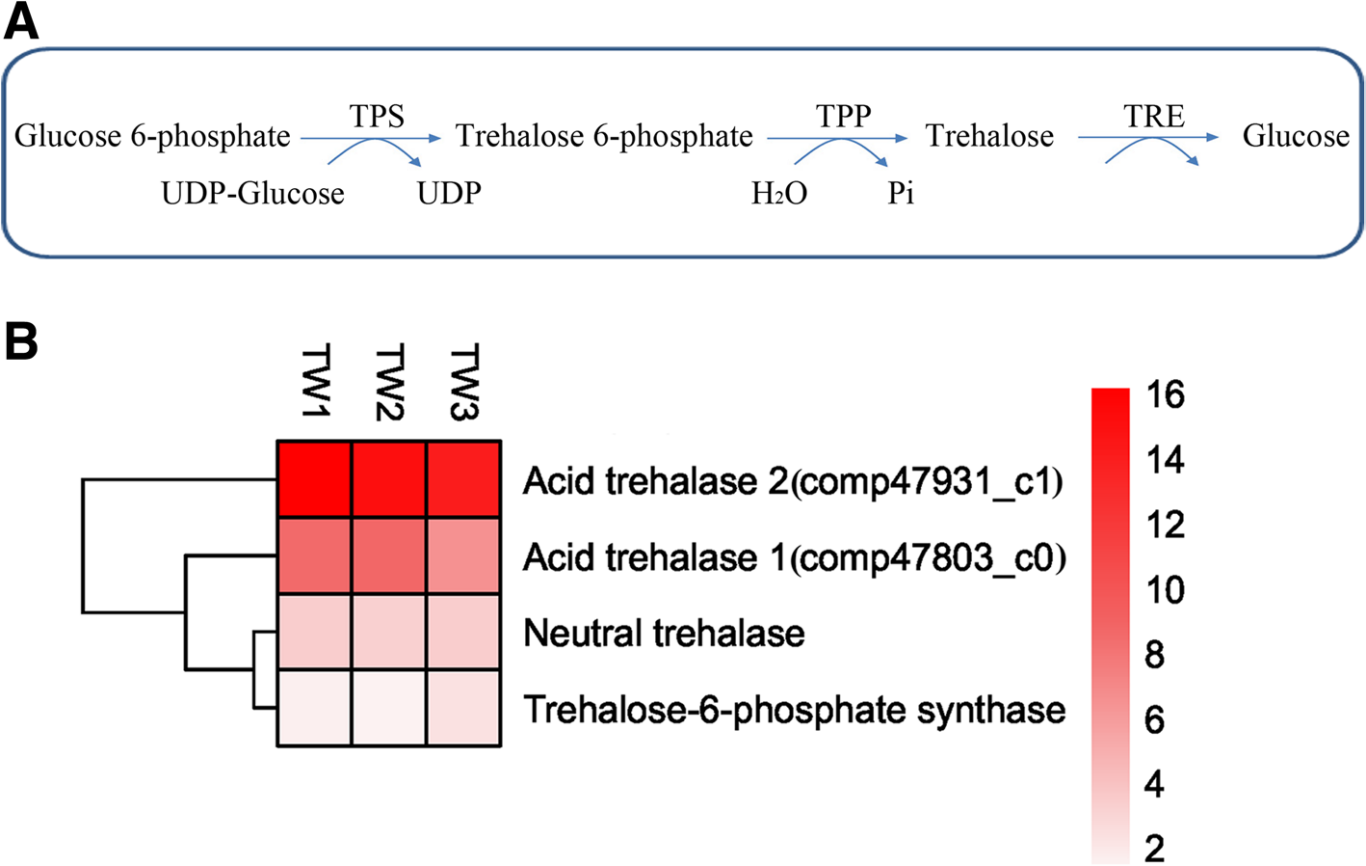
**A** Trehalose metabolic pathway. TPS, trehalose 6-phosphate synthase; TPP, trehalose 6-phosphate phosphatase; TREH, trehalase. **B** Heatmap of FPKM expression values for the annotated genes in *L. luymesi* trehalose metabolic pathway from three individual samples (TW1, TW2 and TW3). FPKM values were listed in Table S4

This study identified four putative genes involved in the trehalose metabolic pathway in *L. luymesi* (Fig. 8, Table S3)*.* One putative *trehalose-6-phosphate synthase* (*TPS*) gene encoded the enzyme that catalyses trehalose synthesis. The deduced *L. luymesi* TPS contains a GT1_TPS domain and a trehalose_PPase domain. At least five different pathways have been described for trehalose biosynthesis in different organisms [23, 68]. However, trehalose is mainly synthesized in eukaryotes by trehalose-6-phosphate synthase (TPS) and trehalose-6-phosphate phosphatase (TPP). TPS catalyses the transfer of glucose from uridine diphosphate glucose to glucose 6-phosphate to generate trehalose 6-phosphate (T6P), whereas TPP catalyses the dephosphorylation of T6P to form trehalose [69–71]. In the present study, we found that the biosynthesis of trehalose in *L. luymesi* is carried out through the TPS/TPP pathway but in a different manner; the process catalysed by a fused gene encoding a protein harbouring conserved TPS/OtsA and TPP/OtsB domains [72], while no TPP gene was identified.

**Fig. 9:**
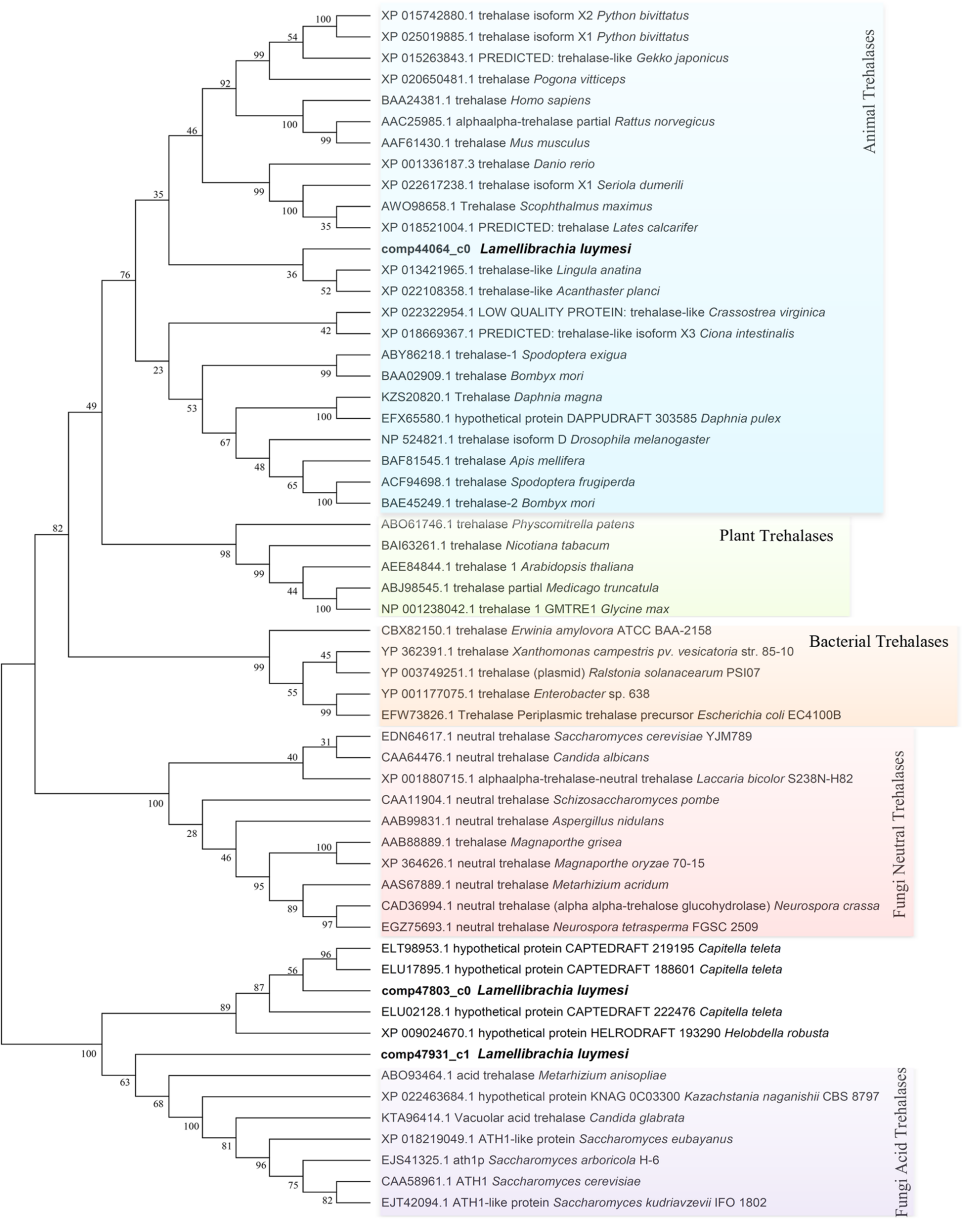
Phylogenetic tree of trehalase genes and protein isoform diversity in *L. luymesi* and other taxa

Compared to trehalose synthesis, hydrolysis of trehalose in *L. luymesi* might be more complex. At least three putative *trehalase* genes (TREs) encode enzymes catalysing the hydrolysis of the sugar. Database searches revealed that the three putative TRE genes encode two forms, namely, neutral (NTH) and acid (ATH) enzymes. Phylogenetic analysis showed that *L. luymesi* NTH was homologous to trehalases from other animals. Moreover, both *L. luymesi* ATHs and fungal ATHs fell into a significant group. The transcript comp47803_c0 (ATH1) with enzymes from other worms further formed a branch, while the unigene comp47931_c1 (ATH2) was assigned another independent branch (Fig. 9).

**Fig. 10:**
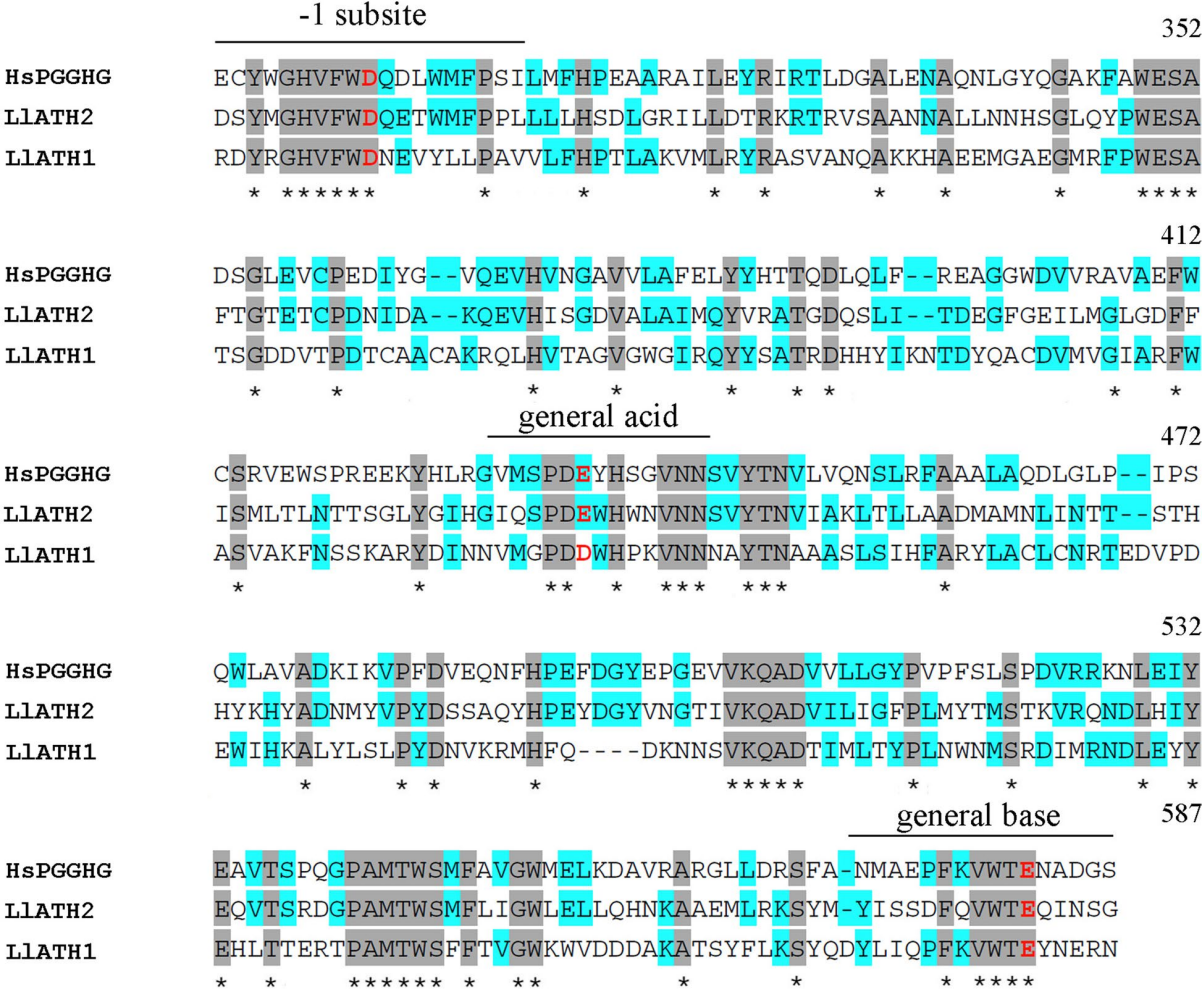
Partial sequence alignment of *L. luymesi* ATHs and human PGGHG. HsPGGHG, *Homo sapiens* PGGHG (NCBI accession no. NP_079368.3); *Ll*ATH1/2, *L. luymesi* acid trehalase 1/2. Identical amino acids are marked by an asterisk and shaded in grey. More than 50% of the conserved residues are shaded in blue. The amino acids in red indicate that three carboxyl residues (corresponding to Asp301, Glu430 and Glu574 of human PGGHG) are essential for catalytic activity

Neutral trehalase is known to hydrolyse the intracellular pool of trehalose in response to physiological responses, whereas the acid enzyme carries out the hydrolysis of exogenous trehalose [73, 74] and might have “scavenger activity” in fungi. Can the presence of multiple trehalase genes be explained by adaptation to a trehalose-rich diet or by the occurrence of different roles of trehalase? Further research is needed to answer this question. However, our sequence analysis found that ATHs in *L. luymesi* might have different enzyme functions. Previous studies have shown that protein-glucosylgalactosylhydroxylysine glucosidase (PGGHG), which effectively releases glucose from type IV collagen, is encoded by the *ATHL1* gene (acid trehalase-like protein), which has been classified into the glycoside hydrolase family 65. Three carboxyl residues (corresponding to Asp301, Glu430, and Glu574 of human PGGHG) were essential for the catalytic activity in this family. The substitution of each of these three residues led to the complete elimination of catalytic activity [75]. Sequence analysis revealed that *L. luymesi* ATHs were members of GH 65, but ATH1 lacks glutamate residues (substitution of Asp) corresponding to human PGGHG Glu430 estimated to function as the catalytic acid, while three carboxyl residues are conserved in ATH2 (Fig. 10). Meanwhile, ATH2 has the highest expression among three trehalases (Fig. 8B).Thus, we speculated that *L. luymesi* ATH2 might have PGGHG activity, and play more important role in cold-seep tubeworm.

## Conclusions

We accomplished high-quality transcriptome assembly of the deep-sea hydrocarbon seep tubeworm *L. luymesi*. We further elucidated the molecular pathways of sulfate activation, cysteine and cholesterol synthesis, and trehalose metabolism. For the first time, two different routes for cysteine biosynthesis described in plants/bacteria and animals were found simultaneously in *L. luymesi*. Moreover, cold-seep tubeworm has the ability of de novo sterol biosynthesis and incorporation and transformation of cycloartenol (plant sterol) and lanosterol (animal/fungi sterol) into unconventional sterols. At the same time, the critical enzymes involved in this process, such as lanosterol 14-alpha demethylase and cycloartenol-C-24-methyltransferase, might have properties similar to those of the enzymes from plants or fungi. Additionally, multiple trehalases might play different roles in cold-seep tubeworms. Contrary to the previous analysis, two pathways for cysteine synthesis and the cycloartenol-C-24-methyltransferase gene were identified in animals for the first time. The present study provides new insights into particular adaptations to chemosynthetic environments in *L. luymesi* and can serve as the basis for future molecular studies on host-symbiont interactions and biological evolution.

## Materials and methods

### Sample collection and RNA extraction

*L. luymesi* were collected in the northern Gulf of Mexico during an August–September 2006 cruise onboard Research Vessel *Seward Johnson* and submersible Johnson Sea-Link I.

Upon arrival at the sea surface, the trophosome tissue samples from three tubeworms were dissected and stored individually in RNAlater (QIAGEN) and used for RNA extraction. The sample was placed into a sterile mortar prechilled with liquid nitrogen. Additional liquid nitrogen was poured onto the tissue sample. A pestle prechilled in liquid nitrogen was used to crack the frozen tissues into a fine powder. TRI Reagent (Molecular Research Center) was then added, and the sample was mixed using a pestle. RNA extraction was performed according to the manufacturer’s protocol. After digestion with RNase-free recombinant DNase I (Takara), RNA was electrophoresed on 1% agarose gels to examine the possibility of RNA degradation and contamination. RNA purity was checked using a NanoPhotometer® spectrophotometer (IMPLEN). RNA concentration was measured using a Qubit® RNA Assay Kit in a Qubit® 2.0 Fluorometer (ThermoFisher Scientific). RNA integrity was assessed using the RNA Nano 6000 Assay Kit of the Agilent Bioanalyzer 2100 system (Agilent).

### Mitochondrial cytochrome c oxidase subunit I gene sequencing

The sample was genotyped by sequencing the mitochondrial gene cytochrome c oxidase subunit I (COI) using previously reported primers [76]. Briefly, genomic DNA was isolated by the phenol‒chloroform DNA extraction method. The extracted DNA was used for PCR amplification. PCR was performed using LA　Taq DNA polymerase (Takara). The PCR program was as follows: 95 °C for 3 min, followed by 35 cycles of 1 min at 95 °C, 1 min at 40 °C and 1 min at 72 °C and 5 min at 72 °C. The PCR products were purified and sequenced. The COI sequences were analysed using NCBI BLAST online (http://blast.ncbi.nlm.nih.gov/Blast.cgi).

### Preparation for Illumina sequencing

Total RNA extracted from the sample was used for sequencing library, which was prepared using the NEBNext® Ultra™ RNA Library Prep Kit for Illumina® (NEB) following the manufacturer’s instructions. Index codes were added to attribute sequences to each sample. Briefly, mRNA was isolated from total RNA using oligo(dT) coupled to magnetic beads. Then, mRNA was fragmented into short fragments using divalent cations under elevated temperature in NEBNext First Strand Synthesis Reaction Buffer (5X). Random hexamer primers were used for the first-strand cDNA synthesis, and the second-strand synthesis was subsequently performed using DNA Polymerase I and RNase H. After exchanging remaining overhangs into blunt ends, the cDNA fragments were adenylated at 3′ ends and ligated with NEBNext Adaptor. Furthermore, the cDNA was purified with the AMPure XP system (Beckman Coulter) to separate fragments of sizes from 150 to 200 bp. Following PCR amplification, purification, and quality control, the cDNA library was constructed.

### Illumina sequencing, transcriptome assembly, and functional annotation

Illumina sequencing, de novo assembly, and functional annotation were performed by Novogene Company Limited, China. Briefly, after the library was qualified, Illumina sequencing was performed using a HiSeq™ 2000 (Illumina) in paired-end mode with a read length of 150 bp. The image data measured by the high-throughput sequencer were converted into sequence data (reads) by CASAVA base recognition. Raw data (raw reads) in fastq format were first processed using an in-house Perl script to acquire clean data (clean reads) by removing reads containing adapters, reads containing N bases, and low-quality reads. Meanwhile, the Q20, Q30 and GC content of the clean data was calculated. All downstream analyses were based on high-quality clean data. The assembly was accomplished using Trinity with min_kmer_cov, which can be set to 2, and all other parameters were set to default values.

Furthermore, the accuracy and completeness of the splicing results were evaluated according to the proportion and completeness of the comparison by BUSCO software. Gene family clustering analysis was performed using Corset (version 4.6) to aggregate transcripts into different clusters based on shared reads. Each cluster was defined as “Gene”. The prefix “comp” was applied to these genes, such as comp10180_c0 and comp10180_c1. All the transcript sequences were searched against protein databases including NCBI Nr (non-redundant) database (e-value < 0.00001), Swiss-Prot (e-value < 0.00001), and KOG/COG (Clusters of Orthologous Groups of proteins) (e-value < 0.001) using BLASTx (Version: BLAST-2.2.28 +). KEGG mapping was performed using KEGG automatic annotation server. Protein domain discovery was performed using the software HMMER 3.0 (hmmscan), with Pfam (Protein family) as the database and an evalue cutoff ≤ 0.01. Gene Ontology terms information for the annotated transcripts was based on the Nr and Pfam annotation using the software blast2go (b2g4pipe_v2.5).

### Sequence alignment and phylogenetic analyses

Characteristic domains or motifs of PAPSS and TPS were identified using a simple modular architecture research tool (SMART) (http://smart.embl-heidelberg.de/). Pairwise and multiple sequence alignments of ATHs were analysed using MAFFT [77]. Multiple sequence alignment of CSs, CYP51s, and trehalases was performed using the ClustalW Multiple Alignment program (http://www.ebi.ac.uk/clustalw/). Phylogenetic tree construction was performed in MEGA 3.1 using the neighbour-joining method [78] with 1000 bootstraps.

## Supplementary Information

**Additional file 1: Figure S1a.** The length distribution of all the transcripts (a) and unigenes (b).

**Additional file 2:  Table S1.**

**Additional file 3:  Table S2.**

**Additional file 4:  Table S3.**

**Additional file 5:  Table S4.**

**Additional file 6.**

## Data Availability

The raw sequencing data have been submitted to the Short Read Archive (SRA) of NCBI under accession number SRR17999638 with Bio project number PRJNA804687 (https://www.ncbi.nlm.nih.gov/bioproject/PRJNA804687/). All data generated or analyzed during this study are included in this published article (and its supplementary information files).

## Abbreviations

TPS: Trehalose-6-phosphate Synthase
TPP: Trehalose-6-phosphate Phosphatase
COI: Mitochondrial Cytochrome c Oxidase Subunit I
PAPSS: Bifunctional 3'-phosphoadenosine-5'-phosphosulfate Synthases
SUL/ATPS: ATP Sulfurylase
KIN/APSK: APS Kinase
FPKM: Fragments per kilobase million
PAP: Adenosine 3’, 5’-bisphosphate
DIDS: 4,4-Diisothiocyanostilbene-2, 2-disulfonic acid
CYS2/cysE: Serine-O-acetyltransferase
CYS3: Cystathionine gamma-lyase
CYS4: Cystathionine beta-synthase
CS: Cysteine synthase
OAS: O-acetylserine
CYP51A1: Lanosterol 14-alpha demethylase/Cytochrome P450 Family 51 Subfamily A Member 1
SRS: Substrate Recognition Sites
SMT: Sterol C24-methyltransferase
T6P: Trehalose 6-phosphate
TRE: Trehalase
NTH: Neutral Trehalase
ATH: Acid Trehalase
PGGHG: Protein-glucosylgalactosylhydroxylysine Glucosidase
