# Supplementary figures and images for: Sulfur, sterol and trehalose metabolism in the deep-sea hydrocarbon seep tubeworm Lamellibrachia luymesi
Source: BMC Genomics. 2023 Apr 5;24:175. doi: 10.1186/s12864-023-09267-8 (PMC10077716; doi:10.1186/s12864-023-09267-8)

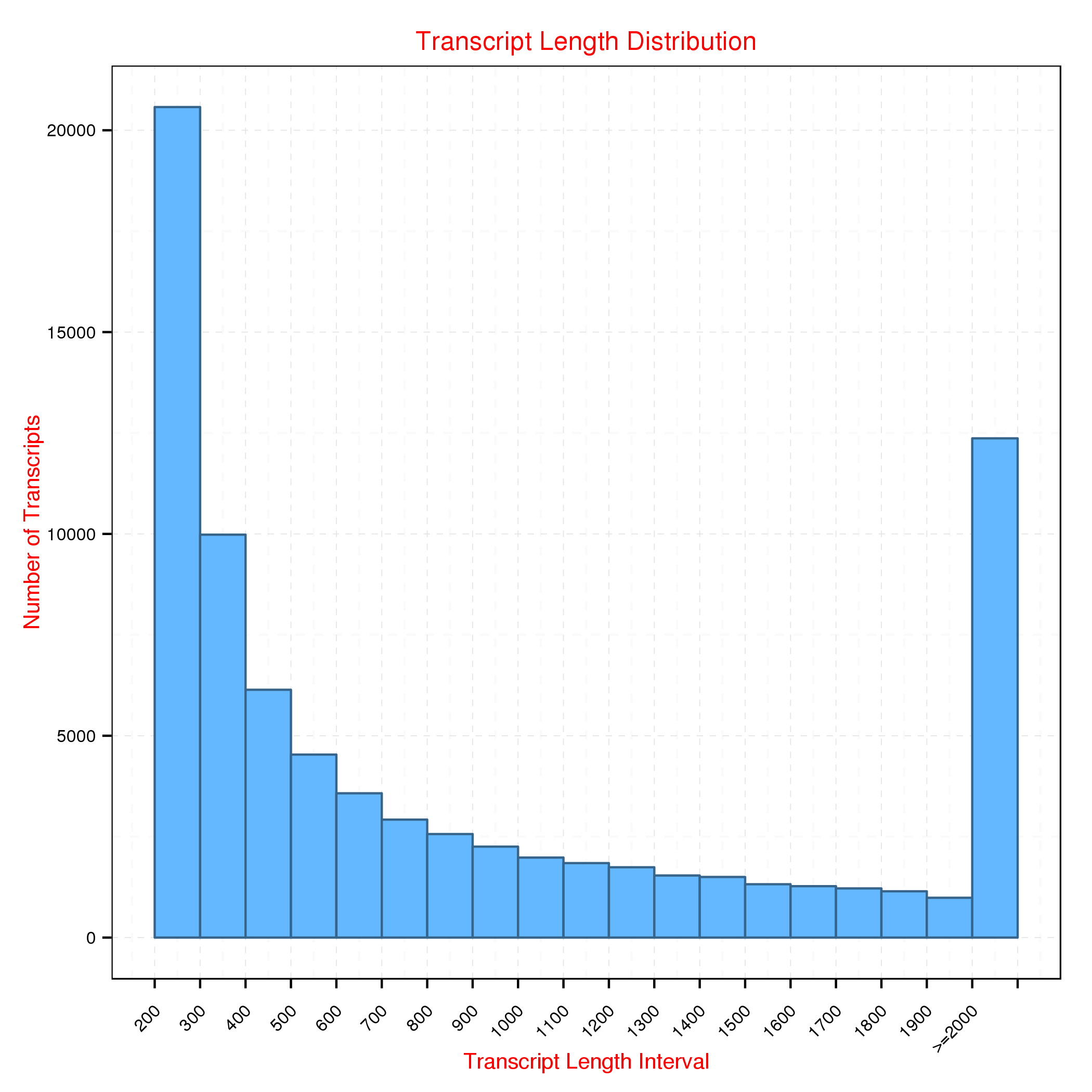

Supplement: Supplementary file 1 — Additional file 1: Figure S1a. The length distribution of all the transcripts (a) and unigenes (b). [file 12864_2023_9267_MOESM1_ESM.zip › Figure S1a.png]

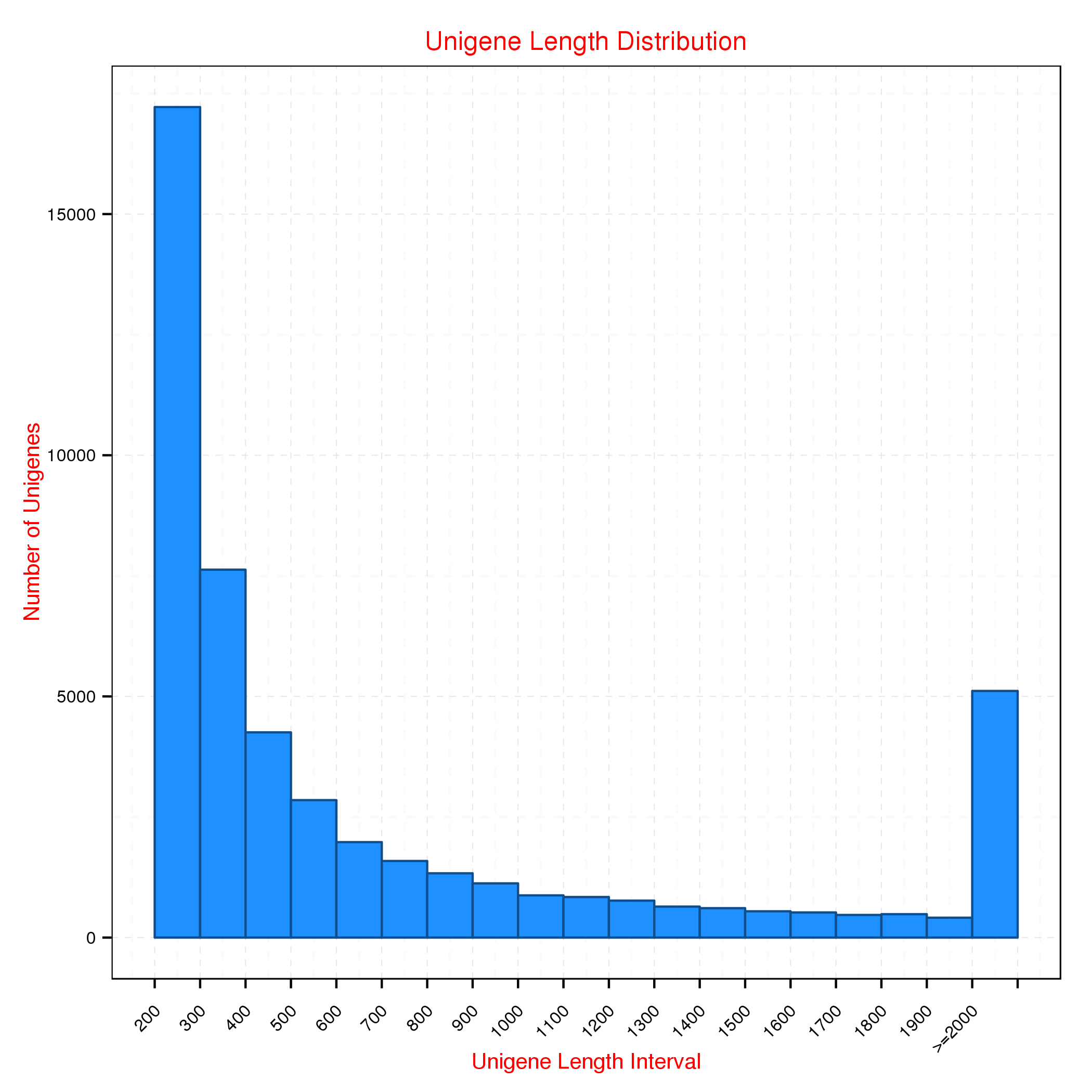

Supplement: Supplementary file 1 — Additional file 1: Figure S1a. The length distribution of all the transcripts (a) and unigenes (b). [file 12864_2023_9267_MOESM1_ESM.zip › Figure S1b.png]
