# Supplementary material for: Sulfur, sterol and trehalose metabolism in the deep-sea hydrocarbon seep tubeworm Lamellibrachia luymesi
Source: BMC Genomics. 2023 Apr 5;24:175. doi: 10.1186/s12864-023-09267-8 (PMC10077716; doi:10.1186/s12864-023-09267-8)
Supplement: Supplementary file 6 — Additional file 6. [file 12864_2023_9267_MOESM6_ESM.docx]

| **No.** | **CYP51 family members in Figure 6&7** | **Organism** | **NCBI accession number** |
| --- | --- | --- | --- |
| 1 | *M.tuberculosis* | *Mycobacterium tuberculosis* | WP_003898577.1 |
| *2* | *M.avium* | *Mycobacterium avium* | WP_009974879.1 |
| 3 | *M.smegmatis* | *Mycolicibacterium smegmatis* | WP_003897264.1 |
| 4 | *M.capsulatus* | *Methylococcus capsulatus* | WP_218800586.1 |
| 5 | *A.thaliana* | *Arabidopsis thaliana* | NP_172633.1 |
| 6 | Potato | *Solanum tuberosum* | XP_006348536.1 |
| 7 | Tomato | *Solanum lycopersicum* | NP_001234537.2 |
| 8 | Rice | *Oryza sativa Japonica Group* | XP_015617432.1 |
| 9 | Wheat | *Triticum aestivum* | XP_044364141.1 |
| 10 | Sorghum | Sorghum bicolor | P93846.1 |
| 11 | *S.cerevisiae* | *Saccharomyces cerevisiae* S288C | NP_011871.1 |
| 12 | *C.elegans* | *Cunninghamella elegans* | Q9UVC3.1 |
| 13 | *U.maydis* | *Ustilago maydis* 521 | XP_011390148.1 |
| 14 | *A.nidulans* | *Aspergillus nidulan* | AAF79204.1 |
| 15 | *F.neoformans* | Filobasidiella neoformans | XP_566464.1 |
| 16 | *C.glabrata* | *[Candida] glabrata* | XP_445876.1 |
| 17 | *C.tropicalis* | *Candida tropicalis* MYA-3404 | XP_002550985.1 |
| 18 | *C.dubliniensis* | *Candida dubliniensis* CD36 | XP_002420370.1 |
| 19 | *C.albicans* | *Candida albicans* SC5314 | XP_716761.1 |
| 20 | *S.pombe* | *Schizosaccharomyces pombe* | NP_592990.1 |
| 21 | *P.digitatum* | *Penicillium digitatum* Pd1 | XP_014532172.1 |
| 22 | *A.fumigatus* | *Aspergillus fumigatus* | AAF32372.1 |
| 23 | *V.nashicola* | *Venturia nashicola* | CAC85409.1 |
| 24 | *V.inaequalis* | *Venturia inaequalis* | AAF71293.1 |
| 25 | *M.graminicola* | *Mycosphaerella graminicola* | AAF74756.1 |
| 26 | *M.fruticola* | *Monilinia fructicola* | AAL79180.1 |
| 27 | *B.fuckeliana* | *Botrytis cinerea* B05.10 | XP_001549961.1 |
| 28 | *M.yallundae* | *Oculimacula yallundae* | AAG44831.1 |
| 29 | *M.acuformis* | *Oculimacula acuformis* | AAF18468.1 |
| 30 | *B.graminis* | *Blumeria graminis* | AAC97606.1 |
| 31 | *U.necator* | *Uncinula necator* | O14442.2 |
| 32 | Mouse | *Mus musculus* | AAF73986.1 |
| 33 | Rat | *Rattus norvegicus* | NP_037073.1 |
| 34 | Pig | [*Sus scrofa*](https://www.ncbi.nlm.nih.gov/protein/NP_999597.1?report=genbank&log$=prottop&blast_rank=1&RID=Y057BEWJ016) | NP_999597.1 |
| 35 | Human | *Homo sapiens* | NP_000777.1 |
| 37 | *Lottia gigantea* | *Lottia gigantea* | XP_009066469.1 |
| 38 | *Capitella teleta* | *Capitella teleta* | ELU17794.1 |
| 39 | *Aplysia californica* | *Aplysia californica* | XP_005103142.1 |
| 40 | *Biomphalaria glabrata* | *Biomphalaria glabrata* | XP_013087940.1 |
| 41 | *Lingula anatina* | *Lingula anatina* | XP_013417877.1 |
| 42 | *Pomacea canaliculata* | [*Pomacea canaliculata*](https://www.ncbi.nlm.nih.gov/protein/XP_025095841.1?report=genbank&log$=prottop&blast_rank=1&RID=Y04UKZPP016) | XP_025095841.1 |
